# Newly reported chloroplast genome of *Sinosenecio albonervius* Y. Liu & Q. E. Yang and comparative analyses with other *Sinosenecio* species

**DOI:** 10.1186/s12864-022-08872-3

**Published:** 2022-09-08

**Authors:** Jing-Yi Peng, Xiao-Shuang Zhang, Dai-Gui Zhang, Yi Wang, Tao Deng, Xian-Han Huang, Tian-Hui Kuang, Qiang Zhou

**Affiliations:** 1 College of Biology and Environmental Sciences, Jishou University, Jishou, 416000 Hunan China; 2 CAS Key Laboratory for Plant Diversity and Biogeography of East Asia, Kunming Institute of Botany, Chinese Academy of Sciences, Kunming, 650201 Yunnan China; 3 Key Laboratory of Plant Resources Conservation and Utilization, Jishou University, College of Hunan Province, Jishou, 416000 Hunan China

**Keywords:** *Sinosenecio*, Comparison of the chloroplast genome, Phylogenetic analysis

## Abstract

**Background:**

*Sinosenecio* B. Nordenstam (Asteraceae) currently comprises 44 species. To investigate the interspecific relationship, several chloroplast markers, including *ndhC*-*trnV*, *rpl32-trnL*, *matK*, and *rbcL*, are used to analyze the phylogeny of *Sinosenecio*. However, the chloroplast genomes of this genus have not been thoroughly investigated. We sequenced and assembled the *Sinosenecio albonervius* chloroplast genome for the first time. A detailed comparative analysis was performed in this study using the previously reported chloroplast genomes of three *Sinosenecio* species.

**Results:**

The results showed that the chloroplast genomes of four *Sinosenecio* species exhibit a typical quadripartite structure. There are equal numbers of total genes, protein-coding genes and RNA genes among the annotated genomes. Per genome, 49–56 simple sequence repeats and 99 repeat sequences were identified. Thirty codons were identified as RSCU values greater than 1 in the chloroplast genome of *S. albonervius* based on 54 protein-coding genes, indicating that they showed biased usage. Among 18 protein-coding genes, 46 potential RNA editing sites were discovered. By comparing these chloroplast genomes' structures, inverted repeat regions and coding regions were more conserved than single-copy and non-coding regions. The junctions among inverted repeat and single-copy regions showed slight difference. Several hot spots of genomic divergence were detected, which can be used as new DNA barcodes for species identification. Phylogenetic analysis of the whole chloroplast genome showed that the four *Sinosenecio* species have close interspecific relationships.

**Conclusions:**

The complete chloroplast genome of *Sinosenecio albonervius* was revealed in this study, which included a comparison of *Sinosenecio* chloroplast genome structure, variation, and phylogenetic analysis for related species. These will help future research on *Sinosenecio* taxonomy, identification, origin, and evolution to some extent.

**Supplementary Information:**

The online version contains supplementary material available at 10.1186/s12864-022-08872-3.

## Background

The sophisticated oxygenic photosynthesis performed by chloroplasts is the most remarkable function of modern plastids. As a photosynthetic organelle capable of supplying energy to green plants, chloroplasts play an important role in photosynthetic oxygen production and secondary metabolism and the biosynthesis of starch, fatty pigments, and amino acids. Chloroplasts and their complex signaling pathways provide a fine regulatory mechanism for plant development, metabolism, and environmental response, forming a major genetic system with the nucleus and mitochondria [1–3].

Chloroplasts also have their independent genomes. Most chloroplast genomes of angiosperms are highly conserved and exhibits a typical quadripartite structure, usually with 110–130 genes, including a large single-copy region (LSC), a small single-copy region (SSC), and two inverted repeat regions (IRs), ranging in size from 120 to 160 kb [4]. Due to its highly conserved nature, slow nucleotide substitution rate, and maternal inheritance, chloroplast DNA, an important information source for taxonomic and phylogenetic research, has been widely used in genomics to research plant phylogeny [5].

*Sinosenecio* B. Nordenstam (1978) (Asteraceae) contains 44 species that are primarily found in central and southwestern China [6–9]. This genus is distinguished by stems that are subscapiform or leafy, palmately or rarely pinnately veined, capitula that range from solitary to numerous, involucres that are ecalyculate or calyculate, and so on. *Sinosenecio* is divided into two species assemblages based on chromosome number and endothecial cell wall thickening patterns, namely the *Sinosenecio s.s*. group and the *S. oldhamianus* group [10–13]. These two groups also differ in geographical distribution. The former is restricted to mountainous regions around Sichuan Basin, southwestern China, and the latter is widely distributed in central and southern China, with two species extending to Indochina.

Previously, several chloroplast markers, including *ndhC-trnV, rpl32-trnL, matK*, and *rbcL*, were used to determine the relationship of *Sinosenecio* species. However, the chloroplast genomes of this genus have not been thoroughly investigated. Here, we sequenced and assembled the chloroplast genome of *Sinosenecio albonervius* Y. Liu & Q. E. Yang. Combined with reported three *Sinosenecio* species (*S. baojingensis* Y. Liu & Q. E. Yang, *S. jishouensis* D. G. Zhang and *S. oldhamianus* (Maxim.) B. Nord) chloroplast genomes, a detailed comparative analysis was carried out in this study.

## Results

### Chloroplast genome basic characteristics of *S. albonervius* and three *Sinosenecio* species

**Fig. 1:**
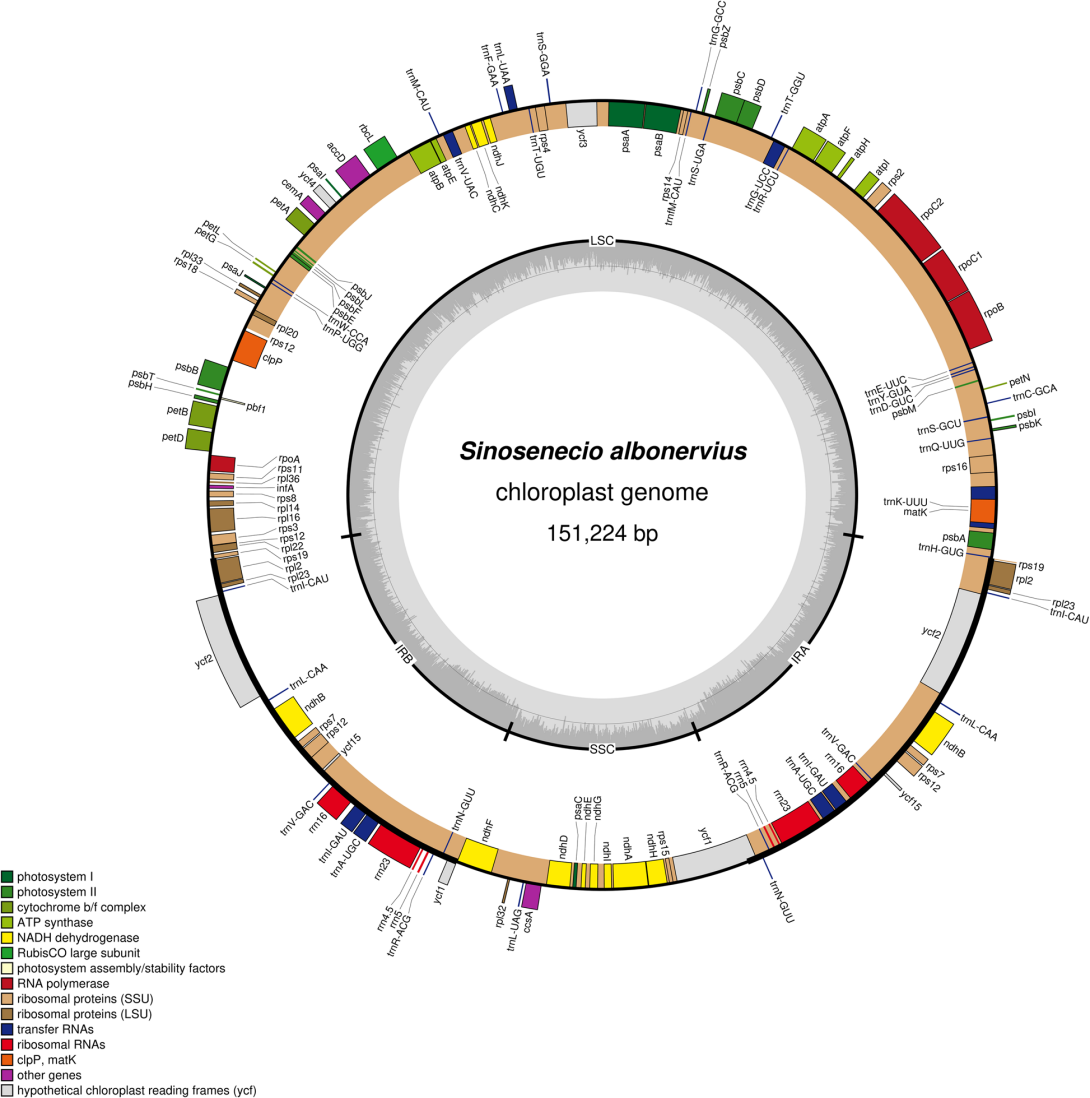
Gene map of the chloroplast genomes of *S. albonervius*. Genes inside the circle are transcribed clockwise, and those on the outside are transcribed counter-clockwise. Genes belonging to different functional groups have been colour-coded. The darker grey area in the inner circle corresponds to GC content, whereas the lighter grey corresponds to AT content

We assembled a 151,224 bp closed circular chloroplast genome with a typical quadripartite structure from the sequencing data of *S. albonervius*, which includes a pair of inverted repeat regions (IRs) of 24,848 bp separated by large single-copy region (LSC) of 83,355 bp and small single-copy regions (SSC) of 18,173 bp (Fig. 1). The sequence of chloroplast genome encodes 134 (two pseudo genes), containing 87 protein-coding genes, 8 ribosomal RNA genes (rRNA) and 37 transfer RNA genes (tRNA) (Table 1). 20 duplicate genes are discovered in the IR regions, with 9 protein coding genes (*rps7*, *rps12*, *rps19*, *rpl2*, *rpl23*, *ycf1*, *ycf2*, *ycf15*, *ndhB*), 4 rRNAs (*rrn16s*, *rrn23s*, *rrn4.5 s*, *rrn5s*), and 7 tRNAs (*trnN-GUU*, *trnR-ACG*, *trnA-UGC*, *trnI-GAU*, *trnI-CAU*, *trnV-GAC*, *trnL-CAA*). 16 genes (*atpF*, *ndhA*, *ndhB*, *petB*, *petD*, *rps12*, *rps16*, *rpl16*, *rpl2*, *rpoC1*, *trnA-UGC*, *trnG-UCC*, *trnI-GAU*, *trnK-UUU*, *trnL-UAA*, *trnV-UAC*) have a single intron, and 2 genes (*ycf3* and *clpP*) contain two introns (Table 2). The overall GC content of this genome is 37.4%, while the corresponding values of the LSC, SSC, and IR regions were 35.50%, 30.60%, and 43.00%, respectively. Additionally, comparison of *S. albonervius* and other *Sinosenecio* species chloroplast genomes was provided (Table 3). The size of chloroplast genomes range from 150,926 to 151,315 bp, of which *S. oldhamianus* is the smallest and *S. baojingensis* is the largest. They have the same number of genes (total genes, protein-coding genes and RNA genes). Moreover, there is no significant difference in GC content between the analyzed genomes.

**Table 1:** The gene composition of *S. albonervius* chloroplast genome, "a" labeled genes have intron

Group of genes	Name of genes
ATP synthase	*atpA, atpB, atpE, atpF*^*a*^*, atpH, atpI*
Photosystem II	*psbA, psbB, psbC, psbD, psbE, psbF, psbH, psbI, psbJ, psbK, psbL, psbM, psbT, psbZ*
NADPH dehydrogenase	*ndhA*^*a*^*, ndhB*^*a*^*, ndhC, ndhD, ndhE, ndhF, ndhG, ndhH, ndhI, ndhJ, ndhK*
Cytochrome b/f compelx	*petA, petB*^*a*^*, petD*^*a*^*, petG, petL, petN*
C-type cytochrome synthesis	*ccsA*
Photosystem I	*psaA, psaB, psaC, psaI, psaJ*
Photosystem biogenesis factor	*pbf1*
Large subunit of rubisco	*rbcL*
Small ribosomal units	*rps11, rps12*^*a*^*, rps14, rps15, rps16*^*a*^*, rps18, rps19, rps2, rps3, rps4, rps7, rps8*
Large ribosomal units	*rpl14, rpl16*^*a*^*, rpl2*^*a*^*, rpl20, rpl22, rpl23, rpl32, rpl33, rpl36*
RNA polymerase sub-units	*rpoA, rpoB, rpoC1*^*a*^*, rpoC2*
Translation initiation factor	*infA*
Ribosomal RNA	*rrn16s, rrn23s, rrn5s, rrn4.5 s*
Transfer RNA	*trnA-UGC*^*a*^*, trnC-GCA, trnD-GUC, trnE-UUC, trnF-GAA, trnG-GCC, trnG-UCC*^*a*^*, trnH-GUG, trnI-CAU, trnI-GAU*^*a*^*, trnK-UUU*^*a*^*, trnL-CAA, trnL-UAA*^*a*^*, trnL-UAG, trnM-CAU, trnN-GUU, trnP-UGG, trnQ-UUG, trnR-ACG, trnR-UCU, trnS-GCU, trnS-GGA, trnS-UGA, trnT-GGU, trnT-UGU, trnV-GAC, trnV-UAC*^*a*^*, trnW-CCA, trnY-GUA, trnfM-CAU*
Acetyl-CoA-carboxylase sub-unit	*accD*
Envelope membrane protein	*cemA*
Protease	*clpP*^*a*^
Maturase	*matK*
Hypothetical genes reading frames	*ycf1, ycf2, ycf3*^*a*^*, ycf15, ycf4*

**Table 2:** Genes with introns in the chloroplast genomes of *S. albonervius* as well as the lengths of the exons and introns

Gene	Location	Exon 1 (bp)	Intron 1 (bp)	Exon 2 (bp)	Intron 2 (bp)	Exon 3 (bp)
*trnK-UUU*	LSC	37	2560	35		
*rps16*	LSC	41	841	214		
*rpoC1*	LSC	432	719	1635		
*atpF*	LSC	145	704	410		
*trnG-UCC*	LSC	23	725	47		
*ycf3*	LSC	124	696	230	740	153
*trnL-UAA*	LSC	37	452	50		
*trnV-UAC*	LSC	38	573	37		
*rps12*	LSC / IR	114	530	232		26
*clpP*	LSC	71	806	291	606	229
*petB*	LSC	6	772	642		
*petD*	LSC	8	718	475		
*rpl16*	LSC	9	1061	399		
*rpl2*	IR	393	667	435		
*ndhB*	IR	777	671	756		
*trnI-GAU*	IR	42	772	35		
*trnA-UGC*	IR	38	821	35		
*ndhA*	SSC	553	1072	539

**Table 3:** Comparison of four *Sinosenecio* species chloroplast genomes

Characteristics	*S. albonervius*	*S. jishouensis*	*S. baojingensis*	*S. oldhamianus*
Accession number	OL678114	NC057061	MZ325394	NC057622
Total length (bp)	151,224	151,257	151,315	150,926
LSC length (bp)	83,355	83,373	83,445	83,092
SSC length (bp)	18,173	18,178	18,172	18,130
IR length (bp)	24,848	24,853	24,849	24,852
Total number of genes	134	134	134	134
Protein coding genes	87	87	87	87
tRNA genes	37	37	37	37
rRNA genes	8	8	8	8
Total GC content	37.4%	37.4%	37.4%	37.3%
GC content in IRs	43.0%	43.0%	43.0%	43.0%
GC content in LSC	35.5%	35.5%	35.5%	35.4%
GC content in SSC	30.6%	30.6%	30.6%	30.6%

### Simple sequences repeats (SSRs) and repeat sequences

**Fig. 2:**
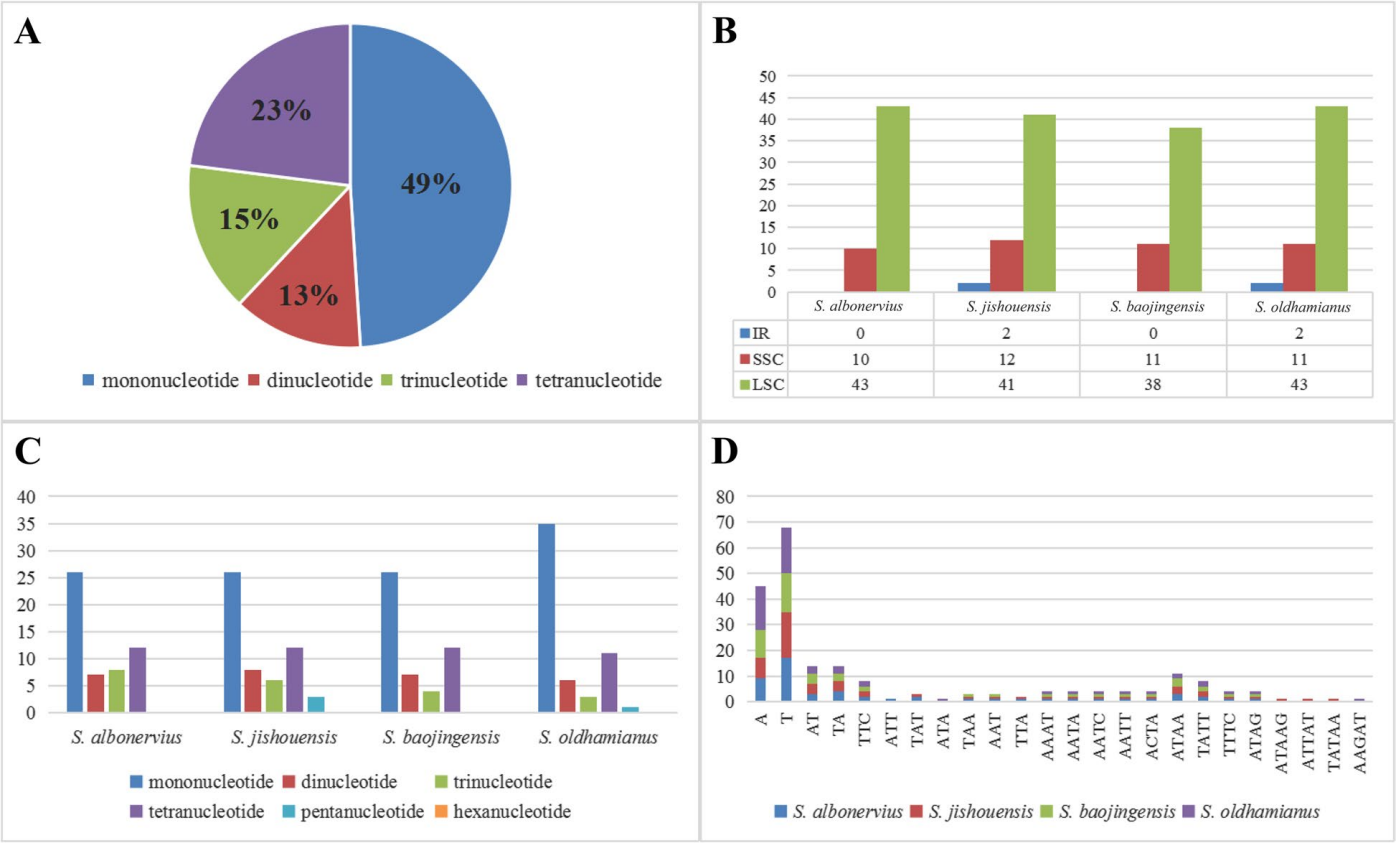
Simple sequence repeats. **A** Proportion of SSR types in *S. albonervius* chloroplast genome. **B** The number of SSRs in LSC, SSC and IRs in *Sinosenecio.* C SSR types in *Sinosenecio*. **D** Specific forms of SSRs in *Sinosenecio*

**Fig. 3:**
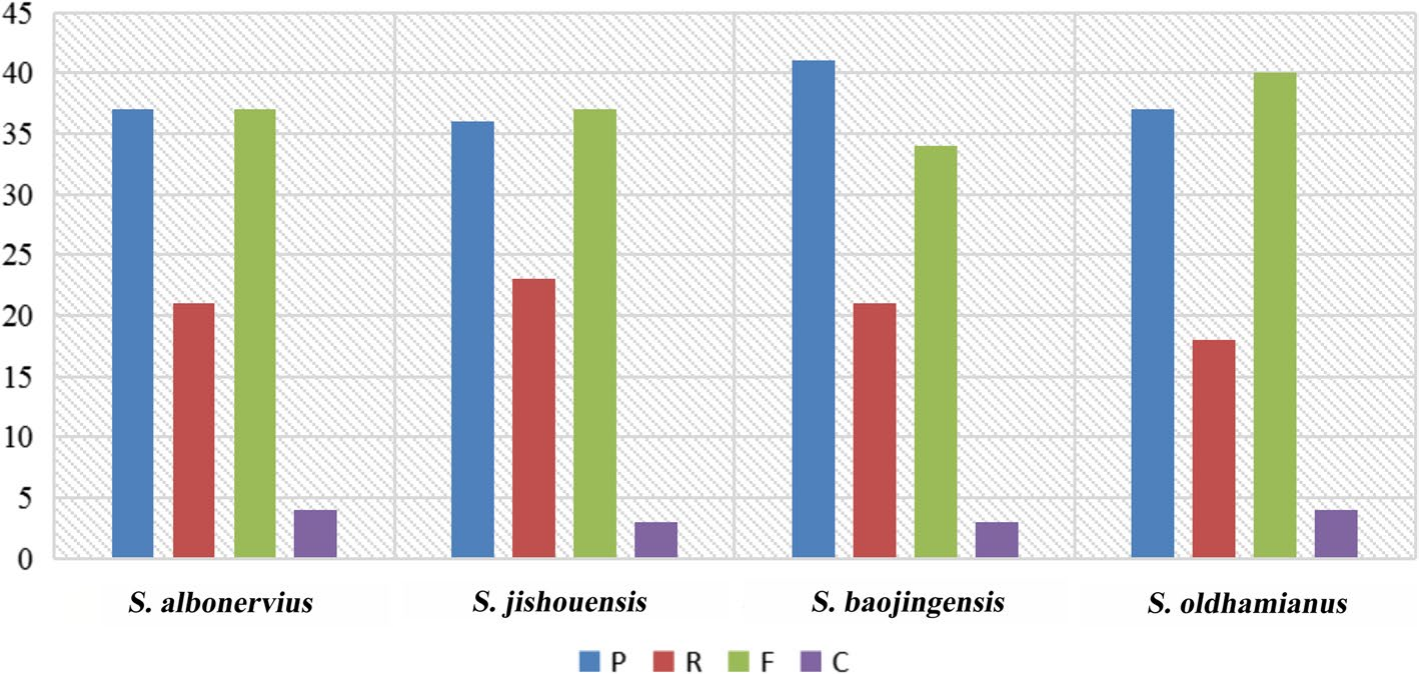
The repeat sequence types in *Sinosenecio*

*S. albonervius* chloroplast genome contained 53 simple sequence repeats (SSRs), including 26 mononucleotide repeats, seven dinucleotide repeats, eight trinucleotide repeats, and 12 tetranucleotide repeats (Fig. 2A). We counted the number of SSRs in SC and IR regions (Fig. 2B) and the different types of SSRs, in each chloroplast genome (Fig. 2C, Table S1). It can be seen that SSRs mainly occur in LSC, while SSRs are not detected in the IR regions of *S. baojingensis* and *S. albonervius*. The SSRs in *S. albonervius*, *S. jishouensis*, *S. baojingensis,* and *S. oldhamianus* are 53, 55, 49, and 56. It is worth noting that mononucleotide repeats of *S. baojingensis* and *S. oldhamianus* are more than the sum of other types. The most common SSRs are mononucleotide repeats composed of A or T (Fig. 2D), and *S. oldhamianus* has the most (35 mononucleotide repeats). In contrast, *S. albonervius* has 26, as do *S. jishouensis* and *S. baojingensis*. Furthermore, we discovered repeat sequences (> 10 bp) in the chloroplast genomes (Fig. 3, Table S2). Palindromic and forward repetitions are more universal than other repetition types. For *S. albonervius*, 99 repeat sequences were identified, which are composed of 37 forward (F), 21 reverse (R), 37 palindromic (P), and four complements (C) repeats, and the largest repeat is a palindromic repeat with a size of 48 bp.

### Codon usage and RNA editing sites

**Fig. 4:**
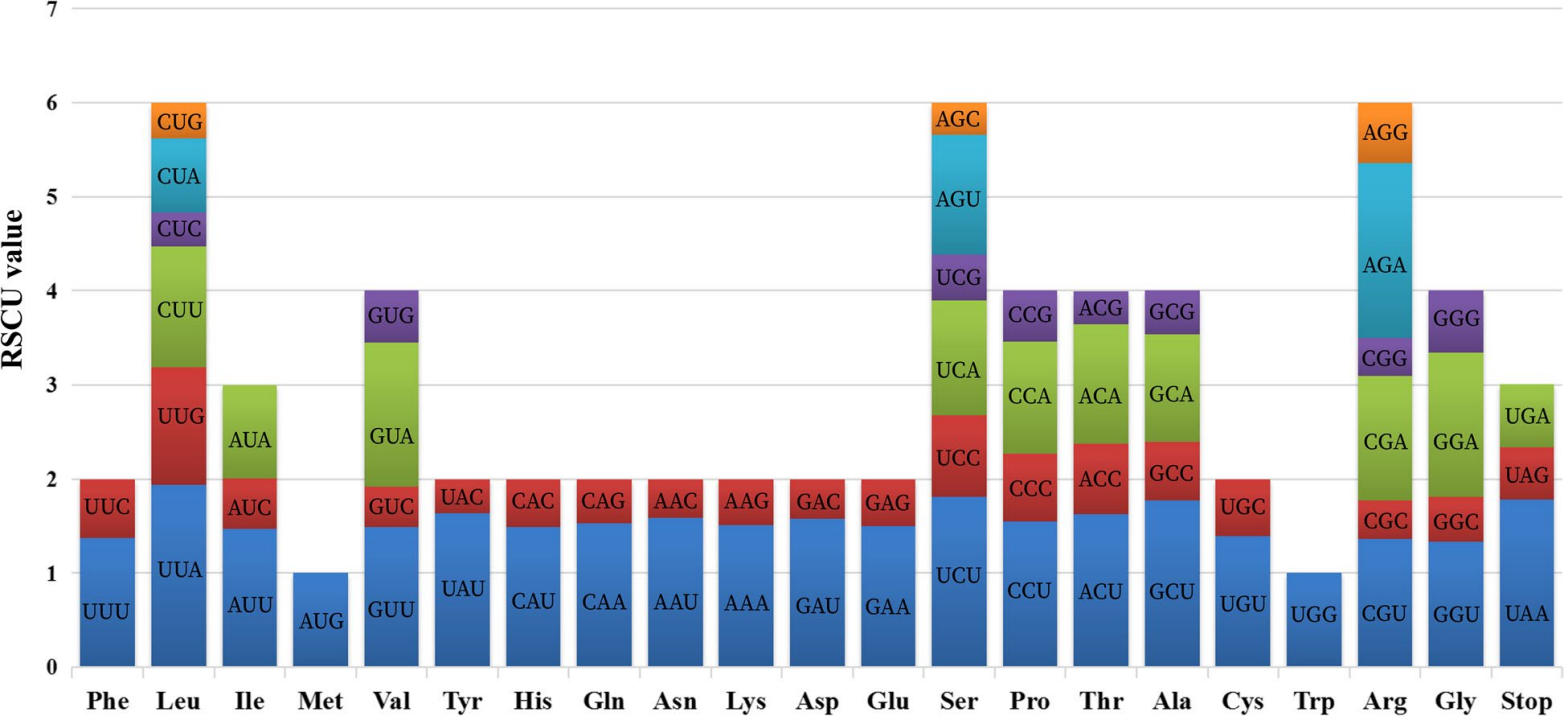
Codon content of amino acids and stop codons in 54 CDS of *S. albonervius*

The codon usage frequency and relative synonymous codon usage (RSCU) frequency were calculated using 54 protein-coding sequences from the chloroplast genome of *S. albonervius* (Table 4). There are 21,301 codons in these protein-coding sequences. With 2281 and 238 codons, Leu and Cys are the most and the least frequently used amino acids respectively. Relative synonymous codon usage analysis (Fig. 4) showed that RSCU value of 30 codons is greater than one, indicating some biased usage for these codons. At the same time, Met and Trp are encoded by a single codon (RSCU = 1), showing no biased usage. Additionally, among the codons with RSCU > 1, only the Leu codon (UUG) is G–ending, and the other 29 codons are A or U–ending.

**Table 4:** Codon usage for *S. albonervius* chloroplast genome by using 54 CDS

Amino Acid	Codon	Number	RSCU	Amino Acid	Codon	Number	RSCU
Phe	UUU	828	1.37	Ser	UCU	478	1.81
	UUC	382	0.63		UCC	231	0.87
Leu	UUA	738	1.94		UCA	324	1.22
	UUG	472	1.24		UCG	126	0.48
	CUU	490	1.29	Pro	CCU	342	1.55
	CUC	136	0.36		CCC	159	0.72
	CUA	301	0.79		CCA	262	1.19
	CUG	144	0.38		CCG	120	0.54
Ile	AUU	897	1.47	Thr	ACU	427	1.63
	AUC	328	0.54		ACC	197	0.75
	AUA	601	0.99		ACA	330	1.26
Met	AUG	518	1		ACG	92	0.35
Val	GUU	424	1.49	Ala	GCU	533	1.77
	GUC	123	0.43		GCC	189	0.63
	GUA	433	1.53		GCA	343	1.14
	GUG	155	0.55		GCG	139	0.46
Tyr	UAU	670	1.64	Cys	UGU	166	1.39
	UAC	148	0.36		UGC	72	0.61
TER	UAA	32	1.78	TER	UGA	12	0.67
	UAG	10	0.56	Trp	UGG	383	1
His	CAU	373	1.49	Arg	CGU	285	1.36
	CAC	128	0.51		CGC	85	0.41
Gln	CAA	594	1.53		CGA	277	1.33
	CAG	180	0.47		CGG	84	0.4
Asn	AAU	830	1.59	Ser	AGU	340	1.28
	AAC	217	0.41		AGC	89	0.34
Lys	AAA	836	1.51	Arg	AGA	389	1.86
	AAG	273	0.49		AGG	134	0.64
Asp	GAU	671	1.58	Gly	GGU	490	1.33
	GAC	177	0.42		GGC	178	0.48
Glu	GAA	834	1.50		GGA	565	1.53
	GAG	275	0.50		GGG	242	0.66

A total of 46 potential RNA editing sites were found in 18 protein-coding genes from the chloroplast genome of *S. albonervius* (Table 5). The *ndhB* genes contain the most RNA editing sites (9 sites), while several genes (*atpI*, *psbf*, *rpl20*, *rpoA*, *rpoB,* and *rps2*) include only one editing site. C-T conversion occurred at the first (21.7%) and second codon positions (78.3%) of all RNA editing sites, indicating that the editing frequency of the third codon position was lower than that of the second or first codon positions. Furthermore, serine codons were edited more frequently than other amino acid codons, and the conversion from serine to leucine occurred the most frequently.

**Table 5:** RNA editing sites in the *S. albonervius* chloroplast genome

Gene Name	Nt pos	AA pos	Align Col	Effect	Score
*accD*	451	151	162	CAC (H) = > UAC (Y)	1
*accD*	824	275	304	UCG (S) = > UUG (L)	0.8
*accD*	1225	409	450	CCA (P) = > UCA (S)	1
*accD*	1433	478	519	CCU (P) = > CUU (L)	1
*atpA*	773	258	258	UCA (S) = > UUA (L)	1
*atpA*	791	264	264	CCC (P) = > CUC (L)	1
*atpI*	629	210	213	UCA (S) = > UUA (L)	1
*ccsA*	110	37	39	CCA (P) = > CUA (L)	0.86
*ccsA*	370	124	127	CCC (P) = > UCC (S)	0.86
*matK*	284	95	108	UCU (S) = > UUU (F)	0.86
*matK*	637	213	229	CAU (H) = > UAU (Y)	1
*matK*	1240	414	430	CAU (H) = > UAU (Y)	1
*ndhA*	566	189	189	UCA (S) = > UUA (L)	1
*ndhA*	1073	358	358	UCC (S) = > UUC (F)	1
*ndhB*	149	50	50	UCA (S) = > UUA (L)	1
*ndhB*	467	156	156	CCA (P) = > CUA (L)	1
*ndhB*	586	196	196	CAU (H) = > UAU (Y)	1
*ndhB*	611	204	204	UCA (S) = > UUA (L)	0.8
*ndhB*	737	246	246	CCA (P) = > CUA (L)	1
*ndhB*	746	249	249	UCU (S) = > UUU (F)	1
*ndhB*	830	277	277	UCA (S) = > UUA (L)	1
*ndhB*	836	279	279	UCA (S) = > UUA (L)	1
*ndhB*	1481	494	494	CCA (P) = > CUA (L)	1
*ndhD*	359	120	128	UCA (S) = > UUA (L)	1
*ndhD*	575	192	200	UCA (S) = > UUA (L)	1
*ndhD*	854	285	293	UCA (S) = > UUA (L)	1
*ndhD*	863	288	296	CCC (P) = > CUC (L)	1
*ndhD*	1286	429	437	UCA (S) = > UUA (L)	0.8
*ndhF*	290	97	97	UCA (S) = > UUA (L)	1
*ndhF*	1340	447	447	UCU (S) = > UUU (F)	1
*ndhG*	166	56	56	CAU (H) = > UAU (Y)	0.8
*ndhG*	314	105	105	ACA (U) = > AUA (I)	0.8
*petB*	418	140	140	CGG (R) = > UGG (W)	1
*petB*	611	204	204	CCA (P) = > CUA (L)	1
*psbF*	77	26	26	UCU (S) = > UUU (F)	1
*rpl20*	308	103	103	UCA (S) = > UUA (L)	0.86
*rpoA*	824	275	279	UCA (S) = > UUA (L)	1
*rpoB*	983	328	345	GCG (A) = > GUG (V)	1
*rpoC1*	511	171	171	CCC (P) = > UCC (S)	1
*rpoC1*	1592	531	548	GCA (A) = > GUA (V)	0.86
*rpoC1*	2039	680	710	CCC (P) = > CUC (L)	1
*rpoC2*	2701	901	1101	CAU (H) = > UAU (Y)	1
*rpoC2*	3695	1232	1452	UCG (S) = > UUG (L)	0.86
*rps2*	248	83	83	UCA (S) = > UUA (L)	1
*rps14*	80	27	27	UCA (S) = > UUA (L)	1
*rps14*	149	50	53	CCA (P) = > CUA (L)	1

### Comparative genomic and nucleotide diversity analyses

**Fig. 5:**
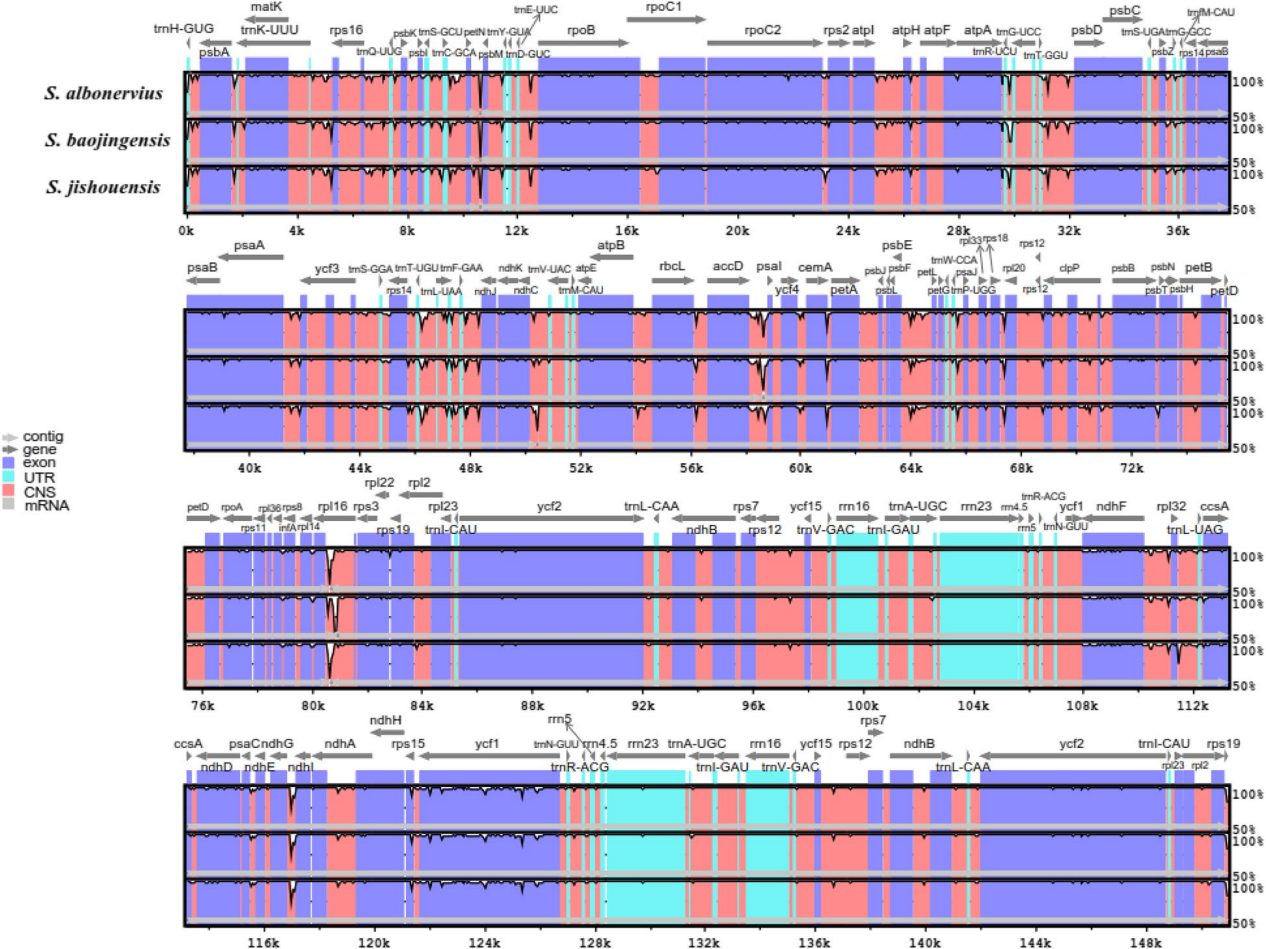
The chloroplast genomes comparison of four *Sinosenecio* species is visualized with *S. oldhamianus* as a reference. The X-axis represents the coordinate in the chloroplast genome. The Y-axis shows different species names, and sequence similarity of aligned regions is displayed as horizontal bars, which expresses as a percentage within 50–100%

**Fig. 6:**
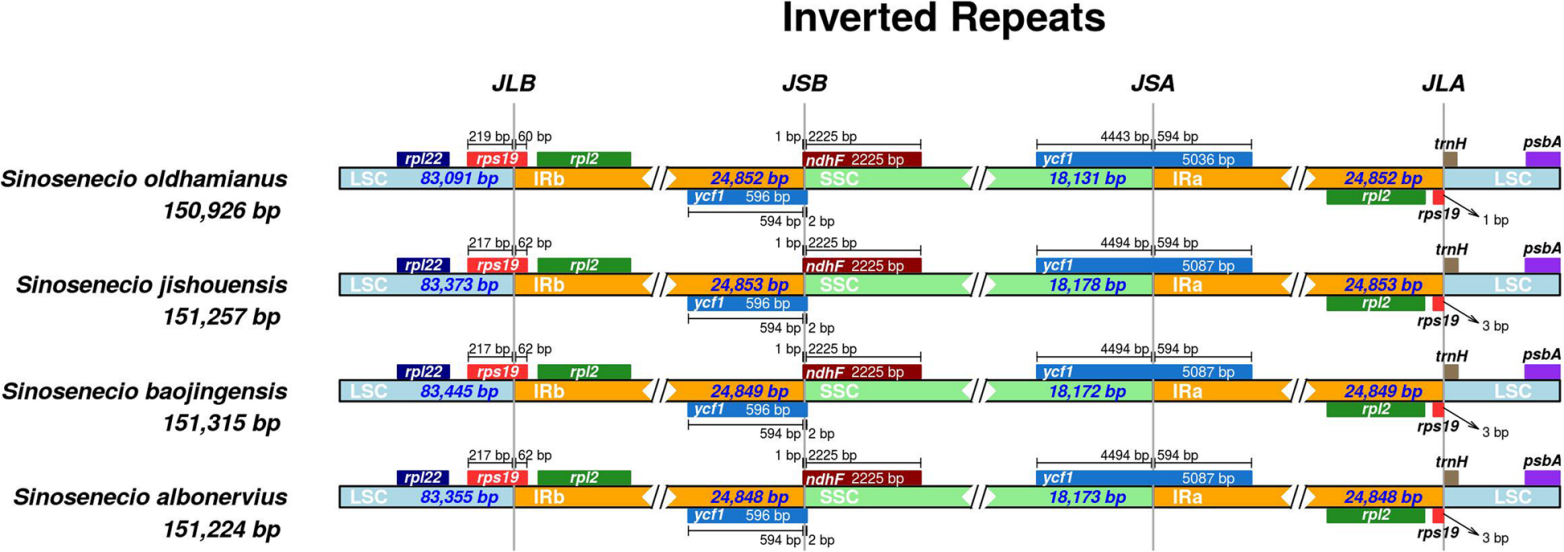
Comparison of connection sites of LSC, IRb, SSC, and IRa in the chloroplast genomes. JLB (IRB/LSC), JSB (IRB/SSC), JSA (SSC/IRA), and JLA (IRA/LSC) represent the junction sites between two adjacent regions in the genome

**Fig. 7:**
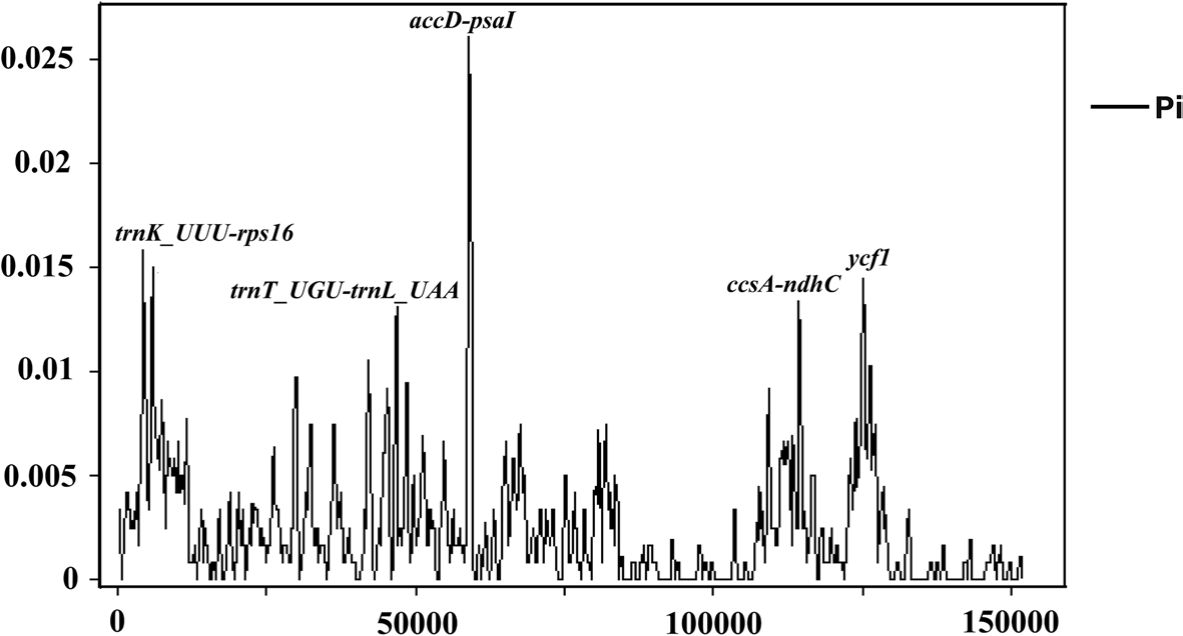
Sliding window analyses of *Sinosenecio* chloroplast genomes using a window length of 600 bp and step size of 200 bp. The nucleotide diversity (Pi) value of each window is shown on Y-axis, and positions are shown on X-axis

The chloroplast genomes of *Sinosenecio* species were compared and analyzed to determine the level of divergence, with *S. oldhamianus* as a reference (Fig. 5). IR regions and the coding regions are more conserved than the SC and non-coding regions. The coding regions of the *ycf1* gene, on the other hand, are the most divergent, with greater diversity than the coding regions of other genes. We also compared IR, SC, and junction sites of *Sinosenecio* species (Fig. 6). The size of IR regions in different chloroplast genomes ranges from 24,848 to 24,853 bp. IR regions contain the *rpl2* gene, three genes *psbA*, *rpl22* and *trnH* in LSC region. SSC/IRa border is located within the coding region of the *ycf1* gene, while *rps19* exists at the junction of LSC/IRb region. Moreover, at JSB, the *ycf1* gene extends into SSC region with 2 bp, and *ndhF* creates a location of 1 bp at the IRb region of each chloroplast genome. The *rps19* gene at JLA extends into SSC region in *S. jishouensis*, *S. baojingensis* and *S. albonervius* with 3 bp, and in *S. oldhamianus* with 1 bp, respectively. DnaSP analyzed the nucleotide diversity to determine the mutation hot spot regions in the chloroplast genome (Fig. 7). Pi values range from 0.00083 to 0.02611. The highest Pi values occurs in *accD–pasI* area with 0.02611, and other high-level peaks (Pi > 0.013) are found in following regions: *trnK_UUU-rps16* (0.01583), *ycf1* (0.01444), *ccsA-ndhD* (0.01333) and *trnT_UGU-trnL_UAA* (0.01306). However, these regions are primarily concentrated in LSC, implying that the LSC contains the most highly diverse regions.

### Phylogenetic analysis

**Fig. 8:**
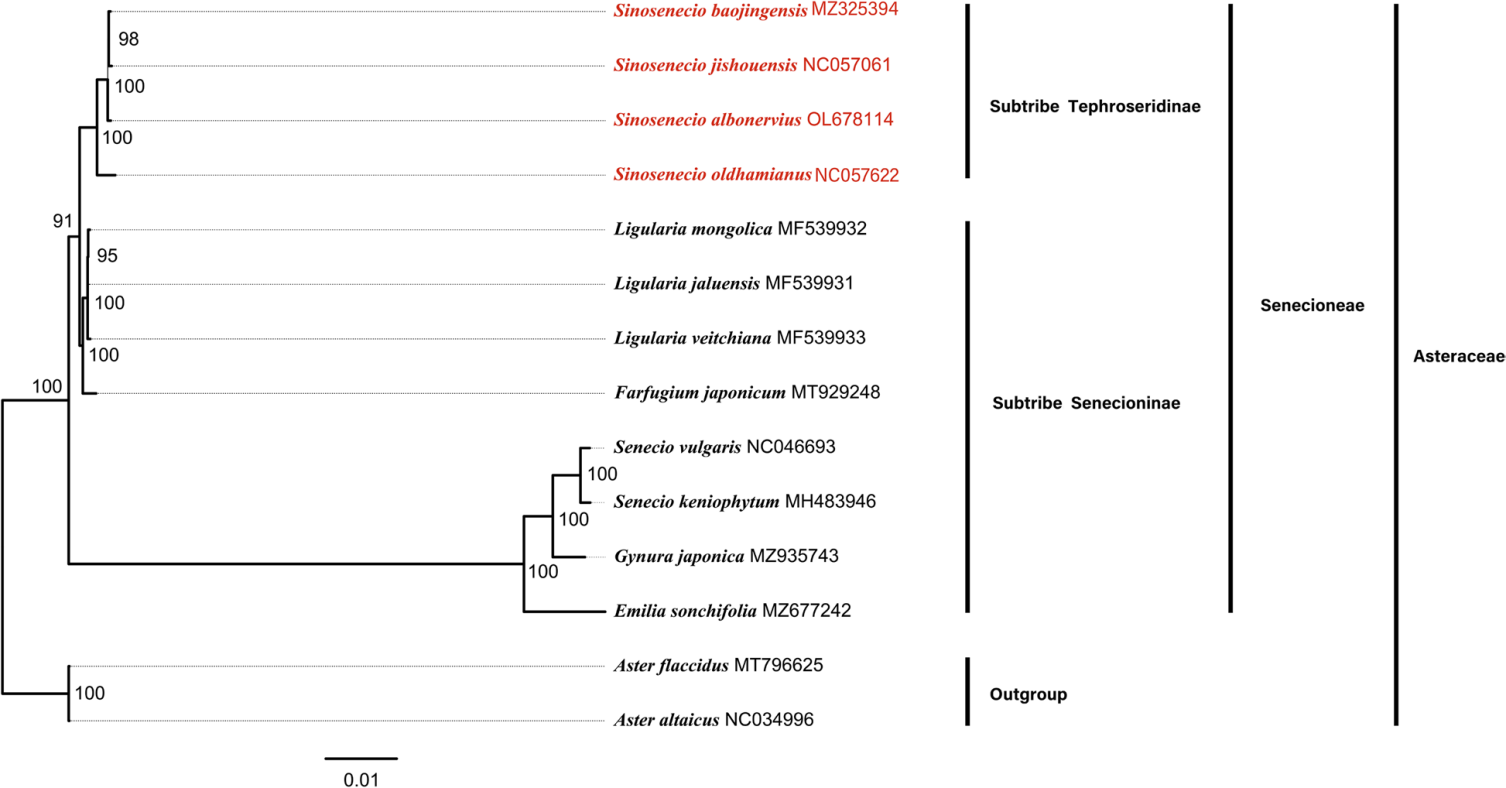
The ML tree based on the chloroplast genomes sequences with GenBank accession numbers. The supported values of each node are shown in this tree, and red fonts indicate the phylogenetic position of *Sinosenecio*

An ML phylogenetic tree was constructed using the chloroplast genome sequence alignments of 14 Asteraceae species (Fig. 8). All nodes have high support values, and Senecioneae of Asteraceae contains three major clades. The first clade includes four species from *Sinosenecio* of subtribe Tephroseridinae and the other two clades consist of eight species from subtribe Senecioninae. In the genus *Sinosenecio*, *S. oldhamianus* is the first to differentiate, followed by *S. albonervius*, and finally *S. baojingensis* and *S. jishouensis*. From the perspective of whole chloroplast genomes, *Sinosenecio* is phylogenetically close to *Farfugium* and *Ligularia*.

## Discussion

### Basic characteristics of *Sinosenecio* species chloroplast genome

We assembled the complete chloroplast genome of *S. albonervius*, and deposited it in Genbank (OL678114). Comparing the chloroplast genomes of *S. albonervius* and the other three *Sinosenecio* species revealed that their genomes have a uniformly typical quadripartite structure with the same numbers of total genes, protein-coding genes and RNA genes as well as consistent GC content. Meanwhile, they differ slightly in the size of the SC and IR regions, which reflects the high degree of conservativeness in angiosperms chloroplast genomes to some extent. 18 genes in *S. albonervius* contain introns that significantly affect RNA stability, regulation of gene expression, and alternative splicing [14]. Additionally, some genes are also sometimes absent from chloroplast genomes of plants. The loss of *rps7* gene is unique to gymnosperms, while the loss of at least seventeen genes (*accD*, *ndhA*, *ndhB*, *ndhC*, *ndhD*, *ndhE*, *ndhF*, *ndhG*, *ndhH*, *ndhI*, *ndhJ*, *ndhK*, *psaJ*, *rpl23*, *rpl32*, *rps15* and *rps16*) was found to be common in angiosperms. However, it is noteworthy that the four *Sinosenecio* species retain the above seventeen genes that are easy to be deleted, and most of these genes are related to NADPH-quinone oxidoreduction [15, 16].

### SSRs and repeat sequences

Simple sequence repeats (SSR) are tandem DNA repeats with short motifs found in plant nuclear, mitochondrial and chloroplast genomes, and exhibit polymorphism and a codominant inheritance pattern. These sequences have been widely used to speculate genetic variation among plant genotypes and as DNA markers in population genetic researches [17, 18]. The SSR abundances in different species are varied [19]. Different numbers of SSR were detected from *Sinosenecio* species chloroplast genomes, while most of the SSRs appear in the SC regions, especially in the LSC region. We found that A or T mononucleotide repetition is the most primary repetitive type, and all mononucleotide repeats are composed of A and T. Such results are consistent with previous reports that A and T are the most abundant repeats in the most angiosperms chloroplast genome, and rarely contain tandem G or C repeats [20]. Furthermore, we discovered 99 repeat sequences in *S. albonervius* chloroplast genomes, the largest of which is a 48-bp palindrome repeat. Repeat sequences are essential genetic resources that play a significant role in phylogenetic studies. Larger and more complex repeat sequences may significantly impact chloroplast genome rearrangement and sequence divergence [21–24].

### Codon usage analysis and RNA editing sites

Synonymous codons encode the same amino acids with different frequencies in many organisms, known as codon bias. The genetic code is usually conserved between organisms but differs in the frequency of codons usage for each amino acid. The selection for which codons are frequent and rare is generally consistent within each genome [25–28]. In our study, the RSCU values of 30 codons are greater than one, indicating a codon bias in the amino acids. Twenty-nine of these codons end in A or T, similar to the codons ending in A/T in most chloroplast genomes, most likely due to the composition bias of the high A/T ratio [29]. The codon usage bias is a common characteristic of eukaryotic genomes and is critical for regulating gene expression [30]. Subsequent research has revealed that RNA editing patterns are a universal phenomenon in higher plants, except the complex leafy licheniformes, a subclass of complex thalloid marchantiid liverworts [31]. It is a process that converts specific RNA nucleotide from C to U and alters the RNA sequence encoded by the genome, but with less frequent conversion from U to C in mitochondria and plastids [32, 33]. In our study, 46 potential RNA editing sites of 18 protein-coding genes in the chloroplast genome of *S. albonervius* were all C-T conversions at the codon's second or third position (21.7 vs. 78.3%). According to previous research, the editing site is usually in the first or second base of codons, resulting in the hydrophilic amino acid being transformed into hydrophobic [1, 32].

### Genomes comparison and nucleotide diversity

We discovered that the chloroplast genomes of *Sinosenecio* species are highly conserved, with high similarity and gene order conservancy. However, the IR and coding regions are more conserved than the SC and non-coding regions, supported by previous findings [34, 35]. The expansion and contraction of boundary regions are evolutionary events and influence chloroplast genomes in size [36]. The length of IR regions ranges from 24,848 to 24,853 bp in *Sinosenecio* genomes. There were two models proposed to explain the extension of the IR regions. Small IR expansion and movement are due to gene conversion, while double-stranded DNA breaks and recombination cause major IR expansion [37, 38]. Furthermore, IRs can stabilize plastomes, and species with IRs in their genomes are more stable in terms of genomic alignment than plastomes lacking one or all IRs [5]. Nucleotide diversity analysis found the hotspot regions for genome divergence, which can be used as new DNA barcodes in species identification [39]. These high Pi loci (*accD–pasI*, *trnK_UUU-rps16*, *ycf1*, *ccsA-ndhD*, *trnT_UGU-trnL_UAA*) are mostly found in the LSC regions. Some of these regions, such as *ycf1*, *ccsA-ndhD*, and *trnT_UGU-trnL_UAA*, have been reported in previous studies on the chloroplast genome [40]. The IR regions are more conserved than SC regions, which may be due to copy correction between IR sequences by gene conversion [41].

### Phylogenetic relationships

The chloroplast genome sequences with sufficient variable loci have been successfully used for classification and phylogenetic studies [42]. To determine *Sinosenecio* phylogenetic relationship, we assembled a dataset of chloroplast genome sequences. The interspecific relationship within *Sinosenecio* has been strongly supported by phylogenetic analysis, and this result is essentially consistent with their taxonomy. However, *Sinosenecio* is a large genus with 44 species, and only four species' chloroplast genome sequences were used in this analysis, making a more comprehensive comparison with phylogenetic results inferred from other chloroplast fragments (*ndhC-trnV*, *rpl32-trnL*) or nuclear genes impossible. In addition, according to Liu 2010, *S. albonervius, S. baojingensis, S. jishouensis*, and *S. oldhamianus*, based on chromosome number and patterns of endothecial cell wall thickenings, were considered to be partial members of *S. oldhamianus* group. This group is closely related to *Nemosenecio* (Kitam.) B. Nord of subtribe Tephroseridinae may represent a new genus or should be merged into *Nemosenecio* [10, 43, 44]. Still, there is not enough molecular data on *Nemosenecio* that we can use to illustrate this conclusion from the level of chloroplast genome at present. Therefore, more taxon sampling and a more rounded analysis of chloroplast genomes are necessary to deeply understand the *Sinosenecio* genetic relationship.

## Conclusions

The complete chloroplast genome of *S. albonervius* was assembled and compared to other *Sinosenecio* species. *Sinosenecio* chloroplast genomes shared structural characteristics such as strict gene order, stable GC content, and relatively conservative IR and coding regions, while boundary region expansion and contraction influence genome size. Some codons encoding amino acids in *S. albonervius* have codon usage bias, which is critical for regulating gene expression. 46 RNA editing sites were detected based on 18 protein-coding genes showing that editing events often occurred in the first and second positions of the codon. Furthermore, the phylogenetic analysis strongly supported the interspecific relationship within *Sinosenecio*, and partial hotspot regions for this genus genome divergence can be used as new DNA barcodes in species identification. Our study provides valuable information for future research on taxonomy, identification, and systematic evolution in *Sinosenecio*.

## Methods

### Plant materials, DNA extraction and sequencing

Fresh *S. albonervius* leaves were collected from Hupingshan Natural Reserve in Hunan Province, China, and dried with silica gel. The voucher specimen was deposited at the herbarium of Jishou University. Plant Genomic DNA Kit DP305 (Beijing, China) was used to extract high-quality total DNA from the silica-dried leaf. Whole-genome sequencing was performed on the Illumina Hiseq platform by Guangdong Mercells Cell Biotechnology Co., Ltd. (Foshan, China).

### Assembly and annotation

The clean data were used to assemble the complete chloroplast genome sequence of *S. albonervius* by the program GetOrganelle [45], and this sequence was annotated on the web page GeSeq (https://chlorobox.mpimp-golm.mpg.de/geseq.html) [46]. The obtained results were checked and manually adjusted in the program Geneious-9.0.2 using *S. jishouensis* as a reference. Finally, the *S. albonervius* chloroplast genome was uploaded to NCBI (Genbank: OL678114). Furthermore, the chloroplast genome map of *S. albonervius* was drawn using the web link (https://chlorobox.mpimp-golm.mpg.de/OGDraw.html) [47].

### Chloroplast genome analysis

The simple sequence repeats (SSR) were detected by using MISA online tool (https://webblast.ipk-gatersleben.de/misa/) [48], and the parameters were set to ten, five, and four repeats for mononucleotide, dinucleotide, and trinucleotide. Three repeats were used for tetranucleotide, pentanucleotide, and hexanucleotide [49]. REPuter was used to analyze forward, palindrome, reverse, and complementary sequences with a minimum repeat length of 10 bp and minimum sequence identity greater than 90% [1, 50].

The expansion and contraction of IR regions in *Sinosenecio* chloroplast genome sequences were studied using the IRscope online program (https://irscope.shinyapps.io/irapp/) [51]. The codon usage of *S. albonervius* chloroplast genome was analyzed using CodonW in MEGA [52], and protein-coding genes with less than 300 nucleotides in length and repeated gene sequences were deleted to reduce the deviation of the results. Besides, the putative RNA editing sites of 18 protein-coding genes were predicted via the PREP-Cp Web server (http://prep.unl.edu/cgi-bin/cp-input.pl), with a cutoff value of 0.8 [53].

*Sinosenecio* chloroplast genomes obtained from Genbank were compared with *S. albonervius* on the mVISTA online program using the Shuffle-Lagan model [54], with *S. oldhamianus* as the reference.

For the nucleotide diversity analysis, *Sinosenecio* complete chloroplast genome sequences were aligned using MAFFT [55]. A sliding window analysis of window length of 600 bp and step size of 200 bp was used in the DnaSP to estimate the nucleotide diversity values [5, 56].

### Phylogenetic analysis

Thirteen complete chloroplast genome sequences, including three *Sinosenecio* species and other ten Asteraceae species sequences, were downloaded from GenBank to clarify the phylogenetic position and relationship of *S. albonervius* with other related species. The genus *Aste*r was selected as an out-group. All these sequences were aligned by using MAFFT, and RAxML-8.2.12 was used for maximum likelihood analysis on Cipres Portal (https://www.phylo.org/portal2) with the GTRGAMMA model, and 1000 bootstrap replicates [57].

## Supplementary Information

**Additional file 1.**

## Data Availability

All the study data used for this analysis can be downloaded from GenBank (accession numbers MF539931 ~ MF539933, MT929248, NC057061, MZ325394, OL678114, NC057622, NC046693, MH483946, MZ935743, MZ677242, NC034996, MT796625). The raw sequence data was uploaded to NCBI SRA database (BioProject: PRJNA783444).

## Abbreviations

IR: Inverted repeat regions
SC: Single copy regions
LSC: Large single copy region
SSC: Small single copy region
SSR: Simple sequence repeats
ML: Maximum Likelihood
